# The Inhibitory Effect of Natural Products on Protein Fibrillation May Be Caused by Degradation Products – A Study Using Aloin and Insulin

**DOI:** 10.1371/journal.pone.0149148

**Published:** 2016-02-16

**Authors:** Eva S. Lobbens, Vito Foderà, Nils T. Nyberg, Kirsten Andersen, Anna K. Jäger, Lene Jorgensen, Marco van de Weert

**Affiliations:** 1 Department of Pharmacy, Faculty of Health and Medical Sciences, University of Copenhagen, Copenhagen, Denmark; 2 Department of Drug Design and Pharmacology, Faculty of Health and Medical Sciences, University of Copenhagen, Copenhagen, Denmark; Consejo Superior de Investigaciones Cientificas, SPAIN

## Abstract

Protein fibrillation is the pathological hallmark of several neurodegenerative diseases and also complicates the manufacturing and use of protein drugs. As a case study, the inhibitory activity of the natural compound aloin against insulin fibrillation was investigated. Based on Thioflavin T assays, high-performance liquid chromatography and transmission electron microscopy it was found that a degradation product of aloin, formed over weeks of storage, was able to significantly inhibit insulin fibrillation. The activity of the stored aloin was significantly reduced in the presence of small amounts of sodium azide or ascorbic acid, suggesting the active compound to be an oxidation product. A high-performance liquid chromatography method and a liquid chromatography-mass spectrometry method were developed to investigate the degradation products in the aged aloin solution. We found that the major compounds in the solution were aloin A and aloin B. In addition, 10-hydroxy aloin and elgonica dimers were detected in smaller amounts. The identified compounds were isolated and tested for activity by means of Thioflavin T assays, but no activity was observed. Thus, the actual fibrillation inhibitor is an as yet unidentified and potentially metastable degradation product of aloin. These results suggest that degradation products, and in particular oxidation products, are to be considered thoroughly when natural products are investigated for activity against protein fibrillation.

## Introduction

Protein misfolding and aggregation are important areas in protein stability research. At present more than 20 incurable diseases, including Alzheimer’s and Parkinson’s disease, are caused by, or associated with, amyloid fibrillation. Yet, fibrillation is not limited to disease-related proteins, and can occur during administration of protein drugs as well. An example is injection amyloidosis, which is characterized by extracellular amyloid deposition at the site of repeated insulin injection in diabetes patients requiring insulin [1–3]. Also in the pharmaceutical industry, fibrillation is problematic, since the occurrence of amyloid fibrils can compromise therapeutic efficacy and patient safety. One of the best-described fibrillation-prone protein drugs is insulin [4;5]. Insulin is a protein hormone used in the treatment of diabetes. The native form of insulin is dominated by α-helical structures and is often formulated as stable zinc-coordinated hexamers mimicking the storage conditions in the pancreas. When administered to the body, the hexamers slowly dissociate into the biological active monomers which are able to regulate the cellular glucose uptake [5;6]. A premature dissociation of the hexamers, for example during manufacturing or storage, followed by partial unfolding of the monomer can induce aggregation and amyloid fibril formation. Upon fibrillation, the protein undergoes structural changes and β-sheet rich structures are formed. Fibrillation kinetics can thus be visualized *in vitro* by adding β-sheet sensitive dyes such as Thioflavin T (ThT) and Congo red [5;7–10]. Several factors are known to affect insulin fibrillation. Among these, agitation, temperature, presence of surfaces or air-water interfaces, presence of stabilizing or destabilizing compounds and low pH can be mentioned. Strategies for stabilization can thus target optimization of the formulation or optimization of conditions during production, storage and delivery of the protein drug [4;5]. In the present study, emphasis was on inhibiting insulin fibrillation through addition of a stabilizing compound. Several natural products have been shown to inhibit amyloid fibril formation [11–14]. In a preliminary unpublished study of different natural products in our lab, the natural compound aloin was found to inhibit insulin fibrillation and selected for further analysis. Aloin is the major anthraquinone glycosyl of the aloe species and exists as a mixture of the two diastereomers aloin A and aloin B. The aloin used for this study primarily consists of aloin A [15–17]. The purpose of this study was to investigate the inhibiting effect of aloin on insulin fibrillation by means of ThT assays and reverse-phase high-performance liquid chromatography (RP-HPLC). However, aloin exhibited an unexpected inhibition profile and several experimental conditions were found to affect the activity of aloin. Further investigation suggested that the activity of the aloin solution was likely caused by an oxidation product formed over several weeks, rather than aloin itself.

## Material and Methods

### Materials

Recombinant human insulin was purchased from SAFC bioscienses (Steinheim, Germany). Aloin (≥ 97%), Thioflavin T (approx. 65%), aloe-emodin, ascorbic acid, formic acid and TFA were purchased from Sigma-Aldrich (Stenheim, Germany). Sodium azide was purchased from Sigma-Aldrich (Switzerland). β-D-(+)-Glucose was purchased from Chromadex (USA). Disodium hydrogen phosphate, sodium chloride and acetonitrile were purchased from Merck Millipore (Darmstadt, Germany). Sodium dihydrogen phosphate, hydrochloric acid and sodium hydroxide were purchased from VWR Int. (Leuven, Belgium). Methanol (HPLC grade) was purchased from LAB-SCAN (Poland). Acetic acid was purchased from Applichem (Darmstadt, Germany). Water was in-house produced, 18 Ω, 0.22 μm.

### Methods

#### Method for dissolving insulin at neutral pH

Before each experiment an insulin stock solution (10mg/mL) was prepared by wetting the insulin with a small amount of diluted PBS buffer (50 mM Phosphate, 0.1 M NaCl, pH 7.4), followed by an addition of small aliquots of 0.1 M HCl until subsequent complete dissolution. The pH was regulated with 0.1 M NaOH until precipitation and complete dissolution occurred. Diluted PBS buffer (50 mM Phosphate, 0.1 M NaCl, pH 7.4) was added to obtain a final insulin concentration of 10 mg/mL. The pH was verified and the solution filtered through a Q-Max 0.2 μm pore cellulose acetate membrane syringe filter with a diameter of 25 mm. The exact concentration was measured on a NanoDrop 2000 UV-Vis spectrophotometer from Thermo Fisher Scientific (USA) by using an extinction coefficient of 1.0 for 1 mg/mL insulin at 276 nm [18].

#### Method for preparing ThT

A ThT stock solution was prepared once every month by dissolving ThT in milli Q water in order to obtain a concentration of 1 mM and then leaving it overnight at room temperature protected from light. The exact concentration was measured on a Cary 100 bio UV-Vis spectrophotometer from Varian (Australia) by using a molar extinction coefficient of 36,000 M^-1^cm^-1^ at 412 nm [19]. The solution was stored at 4°C protected from light.

#### Preparation of aged aloin solution

Aloin stock solutions were prepared by dissolving aloin in 96% ethanol and water in the ratio 1:4 (v/v) in order to obtain a concentration of 20 mM. The aloin solution was stored for 1, 2, 3 or 4 weeks in an Eppendorf tube at room temperature. Control solutions were prepared with ethanol and water alone.

#### Thioflavin T assay at pH 7.4

The ThT assay at neutral pH was performed as described in the study by Groenning et al. [18]. The fibril-sensitive fluorescent probe ThT was used to monitor the fibrillation kinetics of recombinant human insulin in the presence and absence of aloin. In our experience the concentration of ThT does not decrease considerably during storage in a refrigerator for several months; the ThT stock solution was therefore stored for at most one month at 4°C prior to use, while the insulin stock solution (10mg/mL in PBS) was freshly prepared before each fibrillation assay (methods for preparation are described in supporting material). Samples for the fibrillation assay were prepared by mixing aliquots of stock solutions to obtain a final concentration of 1 mg/mL insulin, 50 mM phosphate, 0.1 M NaCl, 20 μM ThT and 0 to 4 weeks old aloin in a concentration of 400 μM unless otherwise stated. Samples in the absence of aloin were prepared with 0.4% ethanol corresponding to the amount of ethanol contributed by the aloin solution. The fibrillation assay was performed in a 96-welled, black, polystyrene, non-sterile plate with optical bottom from Nalge Nunc International (Rochester, NY, USA). In each well a 3.0 mm ± 0.02 mm SiLibeads Typ P glass bead from Sigmund Lindner Gmbh (Warmensteinach, Germany) was added in order to increase the reproducibility and shorten the lag phase. A Polyolefin non-sterile sealing tape from Nalge Nunc International was used to cover the wells to prevent sample evaporation. The ThT fluorescence intensity was measured in a Fluostar Optima plate reader from BMG Labtechnologies (Offenburg, Germany). To avoid interference from the sealing tape, bottom/bottom measurements were performed. The ThT fluorescence was measured using an excitation wavelength of 440 nm and an emission wavelength of 480 nm every 400 seconds. The assay was performed at 40°C with 300 seconds of 1 mm double orbital shaking (corresponding to 600 rpm) per 400 seconds of cycle. At the end of the fibrillation process, the ThT fluorescence was plotted against time and normalized using the following equation: $\frac{y-y_{\text{min}}}{y_{\text{max}}-y_{\text{min}}}$, where y is the measured ThT fluorescence, y_min_ is the lowest ThT fluorescence of the fibrillation curve and y_max_ is the highest ThT fluorescence of the fibrillation curve. In samples where fibrillation was completely inhibited, two of the six replicates were left on the plate reader to obtain a value of complete fibrillation. In these cases y_max_ is the highest ThT fluorescence of the completely fibrillated fibrillation curves.

#### Thioflavin T assay at pH 1.8

A fibrillation assay was performed at pH 1.8 to investigate whether aloin is active against fibrillation when insulin is present in its monomeric form. The samples were prepared as described in the study by Foderà et al. [20]. Briefly summarized, insulin was dissolved in 20% acidic acid and 0.5M NaCl to obtain a final concentration of 1 mg/mL. Then, ThT and 2 weeks old aloin was added to a final concentration of 20 μM and 400 μM, respectively. The fibrillation assay was performed and analyzed as described for the ThT assay at pH 7.4, except that the assay was performed at 45°C without any mechanical shaking of the samples.

#### RP-HPLC analysis of samples from the Thioflavin T assay

The percentage of free residual insulin in the samples was determined by RP-HPLC, after 20 hours of fibrillation assay. Samples were prepared by transferring the content of each well to Eppendorf tubes which were centrifuged for 10 minutes at 14000 rpm at 15°C. The supernatant was analyzed using a 50×4.6mm Kinetex 2.6 μm XB-C18, 100 Å column on a Shimadzu HPLC system from Shimadzu Corp. (Kyoto, Japan). The oven temperature was set to 40°C and mobile phase A consisted of 95% water, 5% acetonitrile and 0.1% trifluoroacetic acid (TFA) while mobile phase B consisted of 95% acetonitrile, 5% water and 0.1% TFA. A flow of 1.85 mL/minute was used. An elution gradient, ranging from 20% B to 50% B from 0 to 3 minutes and from 50% B to 90% B from 3 to 3.5 minutes, was used. The column was then cleaned using an isocratic flow of 90% B from 3.5 to 5.75 minutes and stabilized using an isocratic flow of 20% B from 5.76 to 7.75 minutes. The photodiode array (PDA) detector registered signals in the interval 200–400 nm. The content of insulin in the samples was quantified using a standard curve ranging from 0.01 mg/mL to 1 mg/mL insulin.

#### Transmission electron microscopy

TEM was used to visualize the inhibitory activity of aloin by imaging the fibrils present in the samples after 20 hours of fibrillation assay. Three samples were analyzed, namely a sample containing only 1 mg/mL (172 μM) insulin, a sample containing insulin with 400 μM fresh aloin added and a sample containing a two weeks old 400 μM aloin solution. The imaging was performed on a Philips CM 100 Transmission Electron Microscope using a standard protocol [21;22]. After 20 hours of fibrillation assay, performed as described above, the samples were diluted 50-fold and 3.5 μl aliquots were placed on Copper 400 mesh grids (Agar Scientific, Stansted, UK) coated with Formvar and carbon film. The grid was left for 60 seconds after which 10 μl of distilled water was added and the excess water was removed. Then, 10 μl of 2% uranyl acetate (Agar Scientific) was added and the grid left for 30 seconds. Last, two 10 μl drops of distilled water were placed on the grid, the excess water was removed and the grid was left to dry. [22].

#### Fractionation of aloin sample

RP-HPLC was used to fractionate a two weeks old aloin solution in order to isolate and identify the active compound of the aged aloin solution. 10 μL of the undiluted aged aloin stock solution was injected and fractionated using a 3.0×50mm 1.8 μm Zorbax SB-C18 column on the Shimadzu HPLC system from Shimadzu Corp. (Kyoto, Japan). The oven temperature was set to 40°C and mobile phase A consisted of 95% water, 5% acetonitrile and 0.1% TFA while mobile phase B consisted of 95% acetonitrile, 5% water and 0.1% TFA. The flow was set to 0.2 mL/minute. An isocratic flow of 12% B was used from 0 to 1 minute to stabilize the column. An elution gradient, ranging from 12% B to 60% B from 1 to 18 minutes and from 60% B to 90% B from 18 to 20 minutes was used. Last, the column was cleaned using an elution gradient from 90% to 99% B from 20 to 23 minutes and an isocratic flow of 99% B from 23 to 30 minutes. 0.1 mL fractions were collected corresponding to 30 seconds intervals. The fractionation was performed 18 times and the fractions pooled in appropriate groups based on the peaks of the chromatogram. Furthermore, an additional fractionation was performed, where all fractions were pooled in order to investigate whether the active compound was still present after fractionation. The large fractions were dried in a Heidolph Laborota 4011 digital rotary evaporator (Germany) coupled to a Rotavac valve tec vacuum pump from Heidolph (Germany). The small fractions were dried in a Christ alpha 2–4 LSC freeze drier from Martin Christ (Osterode, Germany) coupled to a Vacuubrand Vacuum pump (Germany). In the freeze drier the pressure was lowered to 1 mbar over a period of 4 hours and a shelf temperature of 20°C was used to avoid freezing the samples.

#### Analysis of the degradation products of aloin by RP-HPLC

The content of the 0, 1, 2, 3 and 4 weeks old aloin stock solution was analyzed by RP-HPLC as described earlier by Ding et al. [17]. The analysis was performed using a 250×4.6 mm 4μm Phenomenex Jupiter 4μ Proteo 90Å C12 column on the Shimadzu HPLC system from Shimadzu Corp. (Kyoto, Japan). The oven temperature was set to 30°C and mobile phase A consisted of 100% water while mobile phase B consisted of 100% methanol. A flow of 0.6 mL/minute was used. An elution gradient, ranging from 45% B to 50% B from 0 to 25 minutes, from 50% B to 65% B from 25 to 55 minutes, from 65% B to 70% B from 55 to 60 minutes, was used. The column was cleaned using an elution gradient, ranging from 70% B to 85% B from 60 to 65 min and an isocratic flow of 90% B from 65 to 70 minutes. The column was stabilized using an isocratic flow of 45% B from 70 to 80 minutes. The PDA detector registered signals in the interval 200–400 nm.

#### Analysis of the degradation products of aloin by LC-MS

LC-MS analyses were carried out using an Agilent 1100 series liquid chromatograph equipped with an autosampler (WPALS G1312A), a degasser (G1379A), a binary pump (G1312A), a column oven set to 40°C (Colcom G1367A), a diode array UV detector SL (G1316A), and coupled to an Agilent MS multimode single quadrupole MS detector (G1956B). 10 μL sample was injected. Separation was performed using the same gradient program, settings and mobile phases as in the RP-HPLC analysis of aloin. The mass spectrometer was run using electrospray ionization in the negative mode as well as the positive mode. Furthermore, a drying gas flow of 9 L/min, a gas temperature of 350°C, a nebulizer pressure of 60 psig, a 150°C vaporizer, and a Vcap of 4000 V was used. A full scan (m/z 300–750) was performed in positive mode and a full scan (m/z 650–750) was performed in negative mode. The samples were analyzed using a fragmentor 80.

#### NMR

For the NMR analyses, aloin was dissolved in deuterated methanol and deuterated water in the ratio 1:4 (v/v) to a concentration of 400 μM. All spectra were acquired using a 600 MHz Bruker Avance III HD equipped with a cryogenically cooled 5 mm dual probe optimized for ^13^C and ^1^H. 600 MHz 1H spectra were obtained with a spectral width of 12 kHz, 32768 data points, 16 scans, with a pulse sequence featuring presaturation of the residual water signal (2 sec.) and composite pulses. 125 MHz 13C spectra were obtained with a spectral width of 36 kHz, acquisition time 0.91 sec., 256 scans and relaxation time 2.45 sec. with a pulse angle corresponding to 30°. Spectra were Fourier transformed to twice the number of data points in the time domain after exponential multiplication with line broadening factors of 0.3 and 1.0 Hz for 1H and 13C spectra, respectively. Spectra were acquired and processed in Topspin (ver., 3.2, Bruker Biospin), and calibrated, normalized and visualized using Matlab (ver. 2015a, The Mathworks, Inc.) using in-house written routines. 1H spectra were calibrated to the position of the HOD-signal set to 4.70 ppm (300 K) and normalized so that the area under the signals in the aromatic range (6.2–7.2 ppm) was equal for all spectra. 13C spectra were calibrated and normalized to the signal from methanol at 49.15 ppm. Signals were assigned by standard NMR-techniques (including HSQC, COSY, HMBC, ROESY) and by comparison with published chemical shifts of aloin and similar structures [17;23].

#### Preparation aloin degradation products

Preparation of aloin B; aloin was dissolved in DMSO (80 mg/mL) and heated at 100°C in a water bath for 10 minutes in order to obtain aloin A and aloin B in the ratio 1:1 [24]. Preparation of 10-hydroxy aloin A and B; aloin was dissolved in ethanol and PBS buffer in the ratio 1:4 (v/v) and stored in an Eppendorf tube at 30°C-35°C overnight [17]. Preparation of elgonica dimers; aloin was dissolved in ethanol and PBS buffer in the ratio 1:4 (v/v) and a CH_2_Cl_2_ phase was added in the ratio 1:1. The solution was shaken for 3 weeks and the CH_2_Cl_2_ phase isolated [17]. The identity of the degradation products was confirmed by either RP-HPLC or LC-MS.

## Results and Discussion

**Fig 1:**
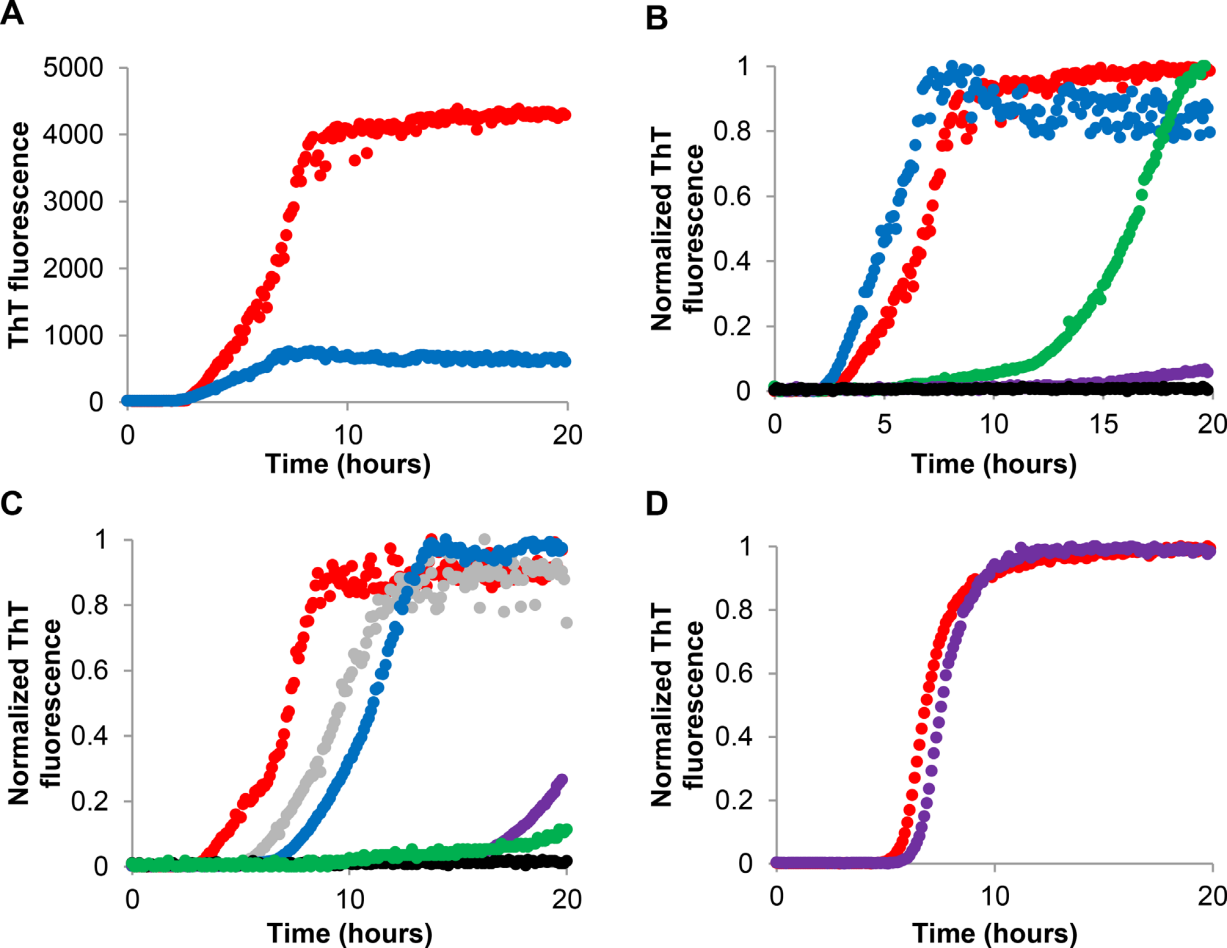
Fibrillation kinetics of insulin incubated in the presence of aged aloin. Representative (B-D normalized) fibrillation curves of 1 mg/mL (172 μM) insulin in the presence of (A) 0.4% EtOH (red), 400 μM fresh aloin (blue), (B) 0.4% EtOH (red), 400 μM aloin stored in solution for 0 (blue), 1 (green), 2 (purple) and 3 (black) weeks, (C) 0.4% EtOH (red), 50 μM (gray), 100 μM (blue), 200 μM (purple), 400 μM (black) or 800 μM (green) 3 weeks old aloin, (D) 0.4% EtOH (red), 2 weeks old aloin (purple). The measurements with EtOH were performed as a control. Fibrillation conditions A-C: ThT assay pH 7.4. Fibrillation conditions D: ThT assay pH 1.8.

**Fig 2:**
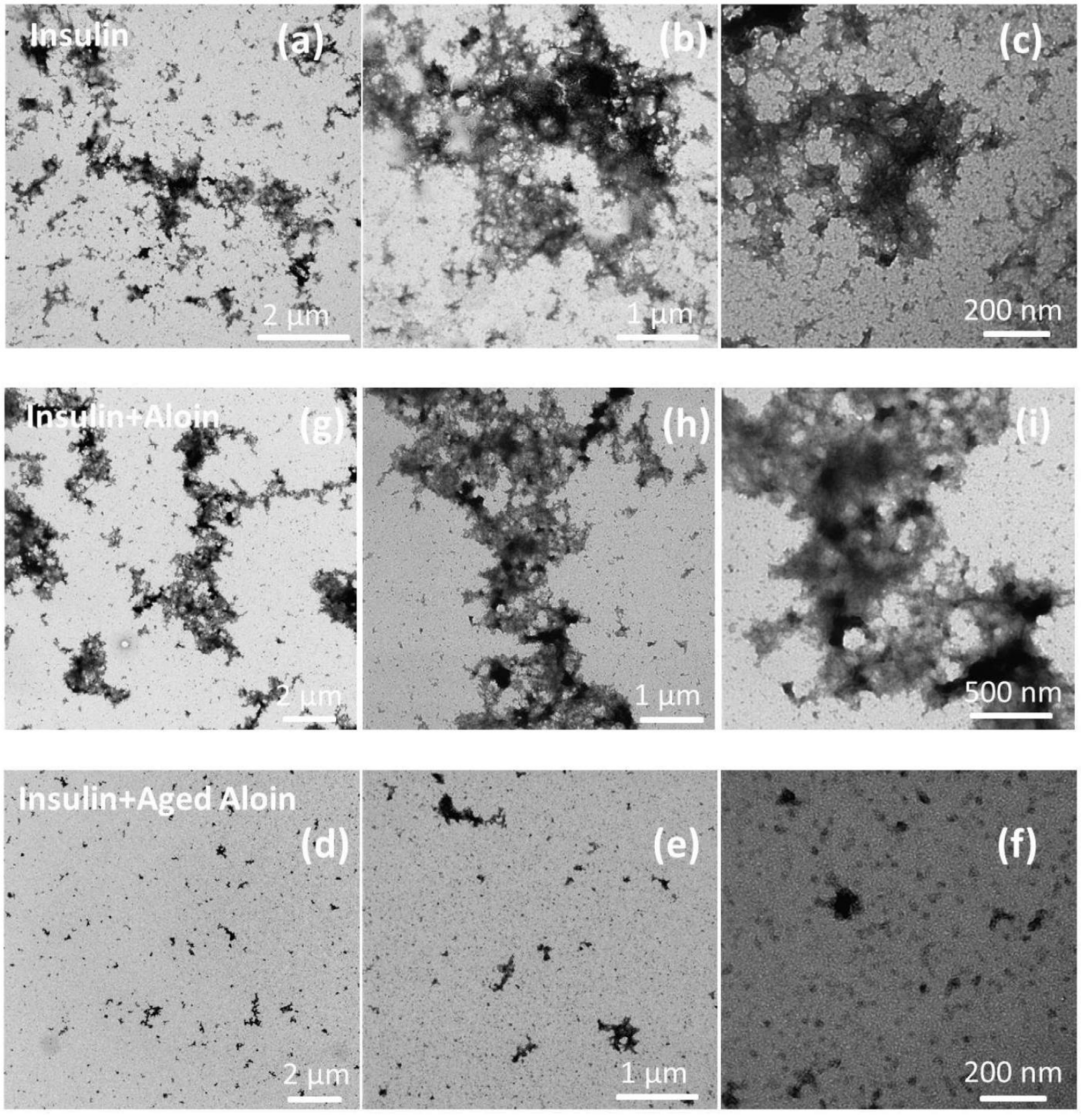
TEM images of insulin incubated in the presence of aged aloin. TEM images of 1 mg/mL (172 μM) insulin after 20 hours of fibrillation assay in the presence of 0.4% EtOH (a-c), 400 μM fresh aloin (g-i) and 400 μM 2 weeks old aloin (d-f). The measurements with EtOH were performed as a control.

The fibrillation kinetics of insulin was monitored by means of the ThT assay. ThT was first introduced in 1959, but is today a widely used dye for amyloid detection. ThT is a fluorescent probe which displays an enhanced fluorescence in the presence of fibril-related β-sheet rich structures and can therefore be used to visualize the three stages of the sigmoidal curve of a typical amyloid fibril formation, namely the lag phase, the elongation phase and the plateau phase [5;25;26]. To investigate the effect of aloin on the fibrillation kinetics, the fibrillation curve of insulin to which an aloin solution was added, was compared to the fibrillation curve of insulin alone (Fig 1). As it is well known that the onset of fibrillation can vary significantly within replicates of the same sample, 6 replicates were performed; however, only one central located curve is depicted here for increased clarity. Figures including all curves are provided in the supporting information (S1A–S2C and S4A–S4D Figs). The maximum fluorescence value was considerably lower for samples containing aloin compared to the sample containing only insulin (Fig 1A). Therefore, all subsequent fibrillation curves are normalized to allow easier visual comparison of the kinetics. The lower fluorescence observed in samples containing aloin could arise from a reduced amount of fibrillated material and the aloin solution could thus be perceived as active. However, HPLC analysis did not show the presence of soluble (monomeric) insulin for the sample containing fresh aloin, despite the clear drop in ThT fluorescence signal. Moreover, TEM analysis did not reveal the presence of non-fibrillar, and thereby ThT-negative, protein aggregates. The lower signal was therefore suggested to be a result of the readings being biased. The fluorescence signal may be biased by the presence of natural compounds with strong absorptive properties, the presence of spectroscopically inactive natural compounds with an ability to compete with ThT for the binding sites, or the presence of compounds with an ability to interact with ThT causing quenching [27]. When analyzing the activity of aloin added to insulin samples at pH 7.4, an increasing activity was observed upon aging of the aloin solution (Fig 1A–1C). Fig 1B shows that aloin dissolved in ethanol (EtOH) and water, and stored for 1–4 weeks at room temperature, prolongs the lag phase considerably, while no apparent difference is found in the elongation phase. The aged aloin solution may therefore primarily affect a process in the lag phase rather than influence the fibrillation process. The activity of the aged aloin solution was confirmed by a RP-HPLC analysis that showed an increased amount of free insulin after 20 hours of fibrillation assay upon addition of the aged aloin solution (Table 1). Only four of the six replicates were tested using RP-HPLC while the remaining two wells were used to obtain a value of complete fibrillation, which was used in the normalization process. Transmission electron microscopy (TEM) analysis of the sample after 20 hours of fibrillation assay showed a considerably decreased amount of aggregates in the presence of a 2 weeks old aloin solution compared to samples in the presence of a fresh aloin solution or without added aloin (Fig 2). This confirms that the insulin fibrillation indeed is inhibited by the aged aloin solution, rather than pushed into an off-pathway aggregation process. The concentration dependence of the activity in the aged aloin solutions was tested on a 3 weeks old solution. Fig 1C shows that aged aloin solutions of 200 μM, 400 μM and 800 μM are able to double the lag phase and thus inhibit insulin fibrillation considerably, while aged aloin solutions of 50 μM and 100 μM only inhibit insulin fibrillation slightly. Finally, we observed that a 2 weeks old aloin solution did not stabilize insulin considerably when the ThT assay was performed at acidic pH, which strongly suggest that the aged aloin solution stabilizes an assembled, multimeric form of insulin, rather than the monomeric form (Fig 1D).

**Table 1:** Percentage of free insulin left after 20 hours of fibrillation assay investigated by RP-HPLC.

Samples	Percentage of insulin left			
	Well 1(%)	Well 2(%)	Well 3(%)	Well 4(%)
Insulin added EtOH	0	0	0	0
Insulin added fresh aloin	0	0	0	0
Insulin added 1 week old aloin	0	2	90	91
Insulin added 2 weeks old aloin	0	98	100	100
Insulin added 3 weeks old aloin	85	100	100	100

Four of the six replicates from the fibrillation assay were tested (well 1–4). Fibrillation conditions: 1 mg/mL insulin (172 μM), 400 μM aloin, 0.4% EtOH.

**Fig 3:**
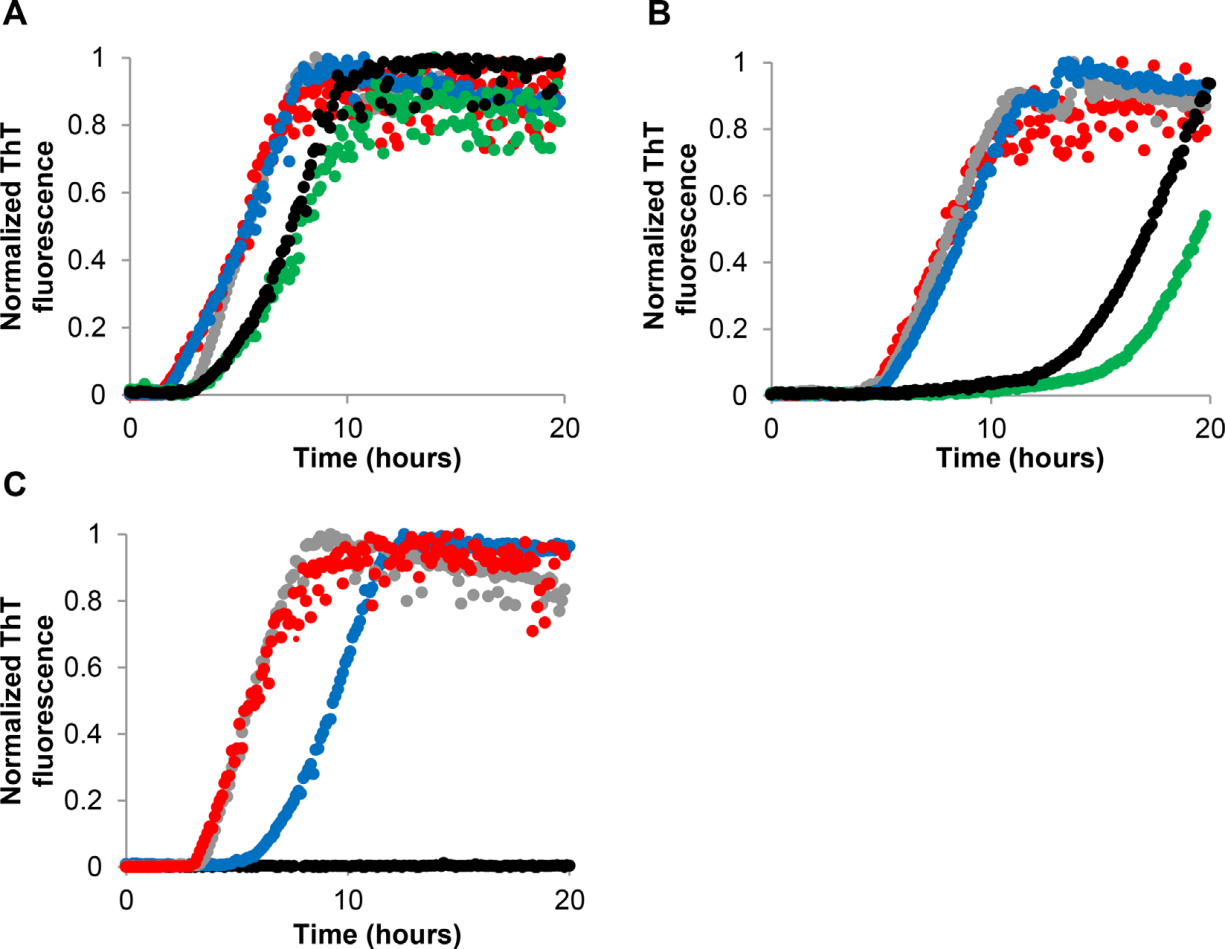
Fibrillation kinetics of insulin incubated with aloin in the presence of anti-oxidants. Representative, normalized fibrillation curves of 1 mg/mL (172 μM) insulin in the presence of (A) 0.4% EtOH (red), 400 μM aloin + 0.1% sodium azide stored in solution for 0 (gray), 1 (blue), 2 (green) and 3 (black) weeks, (B) 0.4% EtOH (red), 400 μM aloin + 2.66 mM ascorbic acid stored in solution for 0 (gray), 1 (blue), 2 (green) and 3 (black) weeks (C) 0.4% EtOH (red), 400 μM aloin stored in the dark for 0 (gray), 1 (blue) and 3 (black) weeks. Fibrillation conditions: ThT assay, pH 7.4.

The increased activity of aloin solutions upon aging was not observed in samples where sodium azide was added (0.1%) to prevent fungal growth (Fig 3A). This observation indicated that the formation of the active degradation product was delayed by the increased amount of nitrogen gas present in the Eppendorf tube, due to degradation of sodium azide. This suggested that the active compound was formed as a result of oxidation of aloin. To investigate this hypothesis, an aloin stock solution containing 2.7 mM ascorbic acid as an antioxidant was prepared, and a new 4 weeks study was performed. The increased activity of aloin upon aging was delayed upon addition of ascorbic acid, which supports the hypothesis that the active degradation product may be an oxidation product (Fig 3B). The potential impact of light on the presumed oxidation of aloin was tested on an aged solution stored in the dark. It was found that the degradation profile of aloin was not altered considerably when the stock solution was stored protected from light (Fig 3C). Oxidation products have previously been shown to affect the inhibitory activity of other tested compounds. In the study by Li et al. [28] and Zhu et al. [29], the inhibitory activity of rifampicin and baicalein against alpha-synuclein fibrillation was suggested to be due to, or enhanced by, an oxidation product which was formed within the first day of incubation. Furthermore, Palhano et al [30] reported that an auto-oxidation product of the natural compound epigallocatechin 3-gallate (EGCG), formed within several hours upon incubation, was likely responsible for remodeling of α-synuclein fibrils into amorphous aggregates. The quinones formed during auto-oxidation of EGCG were also shown to react with free amino groups, e.g. those on the side chain of the amino acid lysine, forming covalent bonds. However, preventing the conjugation of the protein by acylation of the lysine residues did not abolish the activity of EGCG, suggesting a non-covalent interaction is involved in the fibril remodeling [30].

These prior studies thus show that for several inhibitory compounds it is an oxidized degradation product that is the most active compound, and the work on EGCG suggests that the inhibitory activity likely involves non-covalent interactions. Our study on aloin indicates that these oxidized degradation products may develop over much longer time periods than described in the cited studies, depending on the storage conditions and structure.

**Fig 4:**
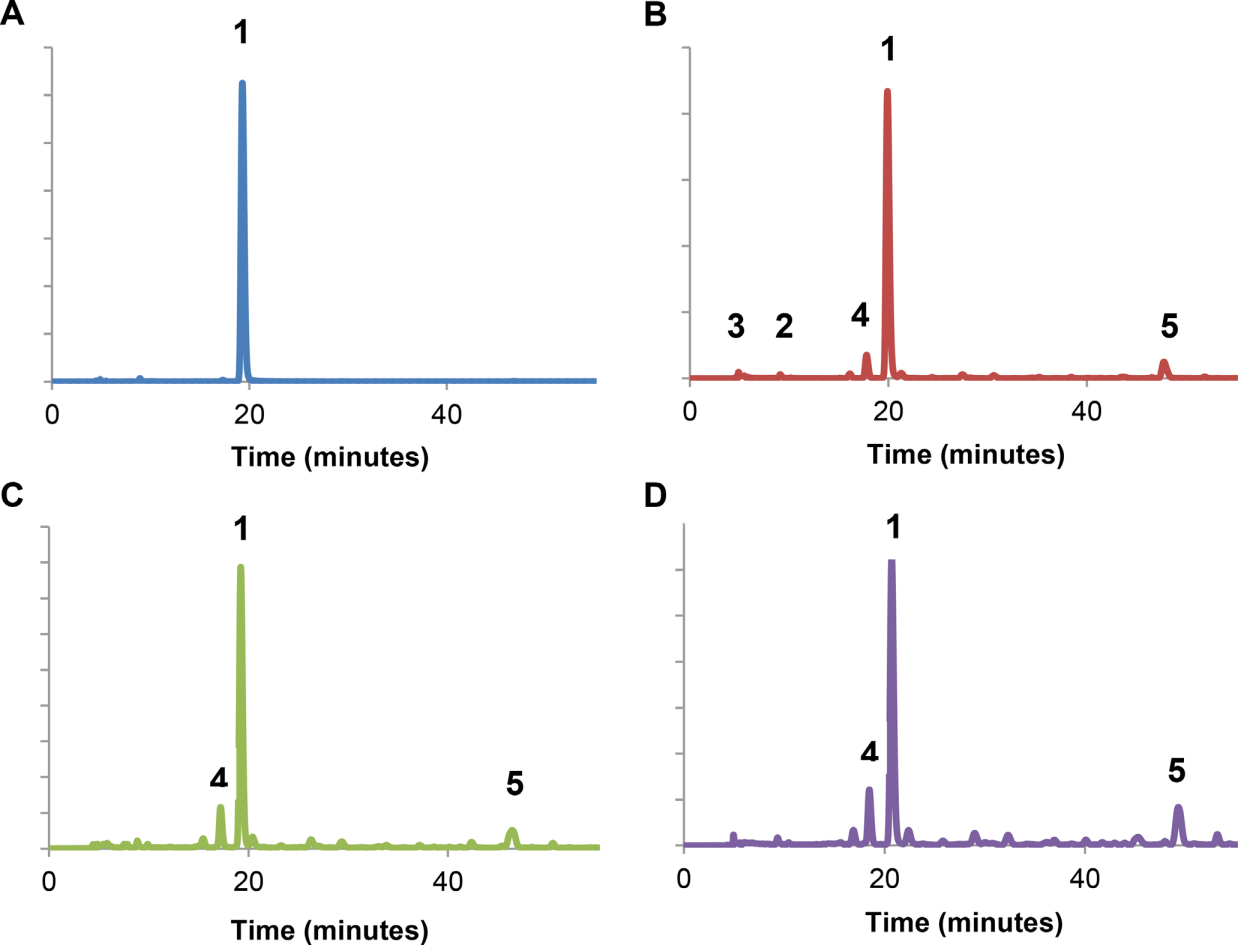
RP-HPLC analysis of aloin stored in solution over time. RP-HPLC analysis of an 20 mM aloin solution stored at room temperature for 0 weeks (A), 1 week (B), 2 weeks (C), 3 weeks (D). Peak assignment: (1) aloin A, (2) 10-hydroxy aloin A, (3) 10-hydroxy aloin B, (4) aloin B and (5) elgonica-dimer. Detection wavelength: 260 nm.

In order to identify the active compound(s), a 2 weeks old aloin solution was fractionated using RP-HPLC. The fractions were dried and reconstituted into samples, each representing a peak in the chromatogram. The reconstituted fractions were screened for activity by performing a ThT assay at neutral pH, but none of the samples showed activity (S3A Fig). Therefore, a ThT assay was performed on aged aloin, dried and reconstituted aged aloin and fractionated aged aloin where all fractions were pooled, dried and reconstituted into a new sample. Surprisingly, none of these samples inhibited insulin fibrillation except for the non-processed aged aloin. This indicates that the activity of aloin was lost during drying of the sample (S3B Fig). Since it was not possible to isolate the active compound by fractionation, the evolution of the degradation products in the aloin solution was studied over time by RP-HPLC and the peaks of the chromatogram assigned to compounds based on the study by Ding et al. [17]. Furthermore, LC-MS was performed on a fresh and a 3 weeks old aloin solution to confirm some of the peak assignments (data not shown). In the HPLC analysis of the fresh aloin solution one major peak was observed around 20 minutes (Fig 4, Peak 1). Based on LC-MS, the peak was assigned to aloin as its mass-over-charge value (*m/z* 419) corresponded to the expected mass of aloin in a mass-spectrum with electrospray ionization in the positive mode. After incubating the aloin solution, several new peaks were observed in the HPLC run. The two first peaks (Fig 4, peak 2+3) were observed with retention times 9 and 10 minutes, and were assigned to 10-hydroxy aloin A and B based on the study by Ding et al. [17]. Furthermore, a new peak was observed at 18 minutes (Fig 4, peak 4). Based on LC-MS, this peak was assigned to aloin B (*m/z* 419, equal to that of aloin A). This result is not unexpected, since it is well known that aloin A converts to the diastereomers aloin B over time. Another small peak was observed after approximately 48 minutes in the HPLC spectrum of the aged aloin solution (Fig 4, peak 5). This more lipophilic compound was tentatively identified as elgonica-dimer based on the expected m/z-ratio (*m/z* 685, electrospray ionization in the negative mode). It was observed that the intensity of the aloin B peak (4) and the elgonica-dimer peak (5) increased upon aging of aloin, while the 10-hydroxy aloin peaks remained at the same level. In addition to these peaks several smaller unidentified peaks were observed in the HPLC spectrum (Fig 4).

**Fig 5:**
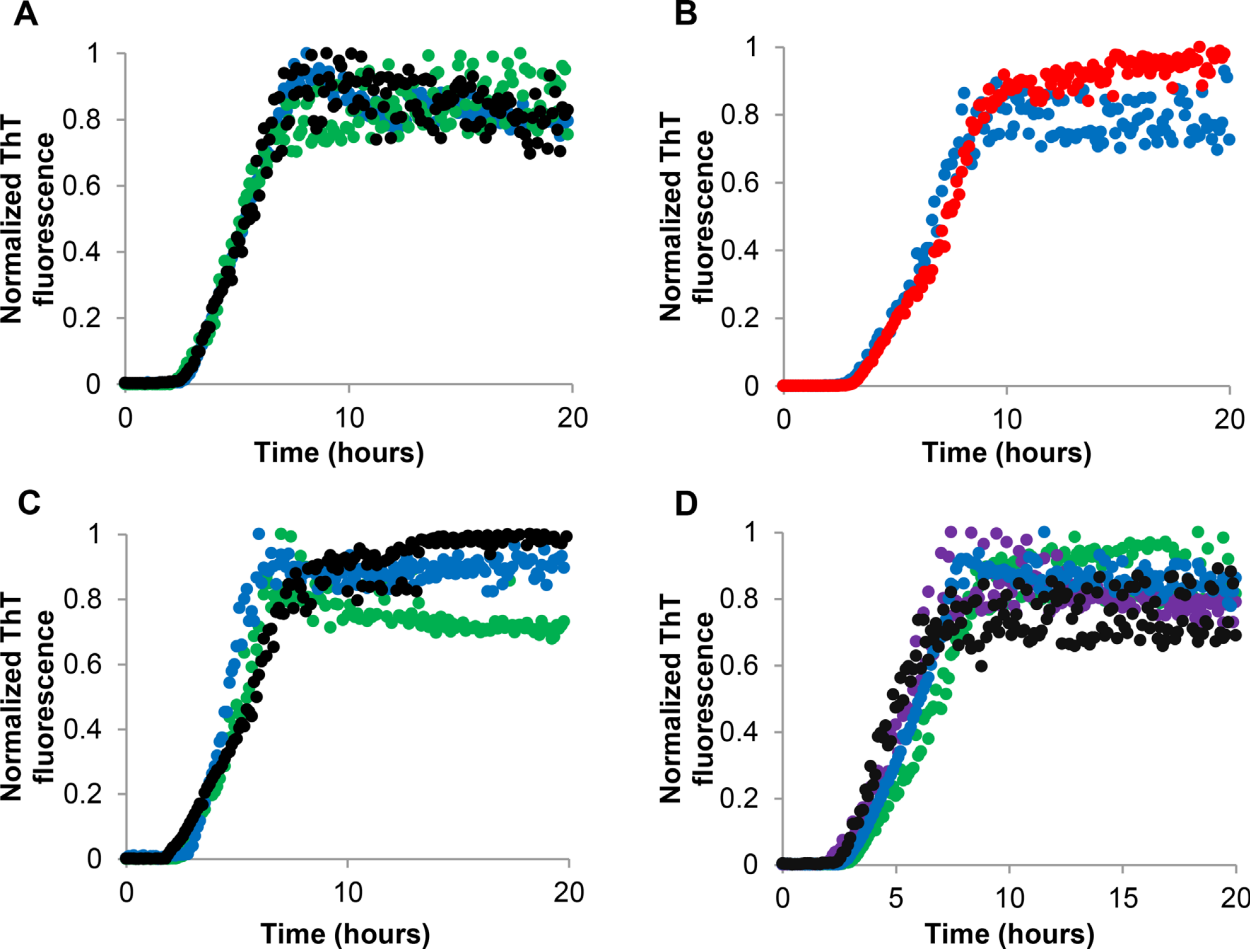
Fibrillation kinetics of insulin incubated in the presence of degradation products of aloin. Representative, normalized fibrillation curves of insulin in the presence of (A) 2% DMSO (green), 400 μM aloin in DMSO (black) and 400 μM aloin heated 10 minutes in DMSO (blue). (B) 0.4% EtOH (red), aloin incubated 1 week in PBS buffer (blue), (C) 2% DMSO (green), 400 μM aloe-emodine (blue) or 400 μM D-glucose (black), (D) 2% DMSO (green), 2% CH_2_Cl_2_ (purple), the CH_2_Cl_2_ phase of a 3 weeks old 400 μM aloin solution incubated with CH_2_Cl_2_ (blue) and the dried CH_2_Cl_2_ phase reconstituted in DMSO (black). Fibrillation conditions: ThT assay, pH 7.4.

Nuclear magnetic resonance (NMR) analyses were performed to investigate whether compounds were produced that were not observable by UV or MS-detection during HPLC analyses. An aloin stock solution was made using deuterated methanol and water (1:4) and ^1^H and ^13^C spectra were obtained during a 4 week period (S5 and S6 Figs respectively). ThT assays were used to monitor the activity (data not shown) and the results revealed a similar increase of activity in the aged aloin solution as the one observed previously in ethanol and water. The NMR analyses revealed that new NMR-signals had appeared in the spectra after one week of storage. The new signals were adjacent to original signals indicating that new compounds had formed, and that these were of the same basic structure as the original. The previously described inversion of the configuration around C-10, forming aloin B from aloin A, could lead to these signals. However, the final number of aloin-like components were more than two as the C-13 signal of C-1’ (Glc C-1) clearly shows at least three signals (S5D Fig). The ^1^H NMR-spectra also shows the formation of several components and a considerable broadening of the original signals. This might indicate the formation of dimers by oxidative coupling of C-10 just as described for dianthrone sennosides [31]. The reduction of the area under the ^1^H signal over time, to approximately 1/3 at week 4 compared to the initial area, also supports this (S6E Fig). The main known and observed degradation products were produced by different means and their activity determined based on ThT assays at neutral pH. First, aloin was boiled in dimethyl sulfoxide (DMSO) creating an aloin solution containing aloin A and aloin B in the ratio 1:1 [24]. The increased amount of aloin B in the solution did not prolong the lag-phase considerably, which indicates that the conversion into aloin B is not responsible for the increased activity of the aged aloin solution (Fig 5A). Aloin was then incubated in PBS at 35°C in order to promote the formation of 10-hydroxy aloin A and B [17]. The presence of 10-hydroxy aloin A and B did not prolong the lag phase considerably, which suggests that the conversion into 10-hydroxy aloin is not responsible for the increased activity of the aged aloin solution (Fig 5B). Aloe-emodine and D-glucose were purchased and tested. Also here, the lag phase was not prolonged considerably, which indicates that a degradation of aloin into aloe-emodine or D-glucose is not responsible for the increased activity (Fig 5C). Finally, a CH_2_Cl_2_ phase was added to the aloin solution in order to isolate the elgonica-dimers during the aging process. After 3 weeks the CH_2_Cl_2_ phase, which was shown to contain mainly elgonica-dimer by LC-MS, was tested in a ThT assay. No activity was found in the CH_2_Cl_2_ phase. To ensure that the inactivity was not due to a solubility issue, the CH_2_Cl_2_ phase was evaporated and the elgonica-dimer dissolved in DMSO and tested in a ThT assay, again failing to show any inhibitory effect (Fig 5D).

**Fig 6:**
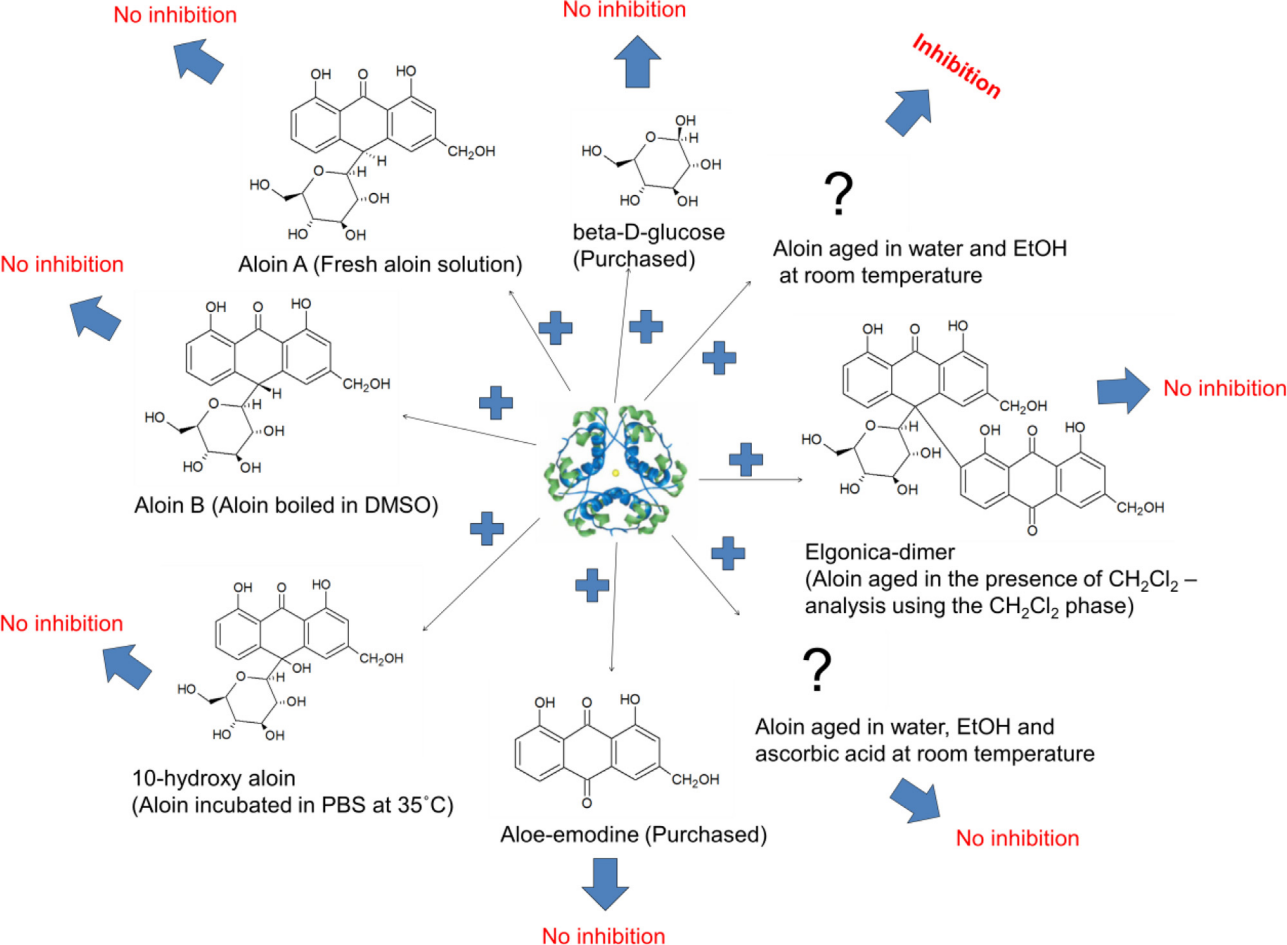
Overview of the activity of the tested degradation products of aloin against insulin fibrillation.

Summarizing the results (illustrated in Fig 6), the inhibitory activity of the natural product aloin against insulin fibrillation was caused by a degradation product, likely formed through an oxidation process, of which the formation was dependent on the incubation conditions. Several degradation products were identifiable in the aged aloin solution, but none of these were found active. Furthermore, the loss of activity of the pooled fractions during freeze-drying indicates that the active degradation product of aloin is metastable. Our studies shows that degradation of a proposed inhibitory compound, and in particular oxidation, is to be considered thoroughly when natural products are chosen as lead compounds against protein fibrillation, since their activity may be altered during production, handling or storage of the product. The activity of oxidation products has previously been shown to affect the activity of tested compounds; however, our results indicate that these oxidized degradation products may develop over much longer time periods (weeks vs hours) than previously described. The apparent important role of oxidation processes in fibrillation inhibition is intriguing, as several known inhibitors are natural compounds with antioxidant properties (e.g. quercetin, EGCG, and baicalein). Unfortunately the mechanism by which the oxidized compound exhibits (higher) inhibitory activity remains as yet elusive. Further in-depth studies are therefore required, as this may provide important insight into the structural requirements for good inhibitors.

## Supporting Information

S1 Fig Fibrillation kinetics of insulin incubated in the presence of aged aloin. Fibrillation curves of 1 mg/mL (172 μM) insulin in the presence of (A) 0.4% EtOH (red), 400 μM fresh aloin (blue), (B) 0.4% EtOH (red), 400 μM aloin stored in solution for 0 (blue), 1 (green), 2 (purple) and 3 (black) weeks, (C) 0.4% EtOH (red), 50 μM (gray), 100 μM (blue), 200 μM (purple), 400 μM (black) or 800 μM (green) 3 weeks old aloin, (D) 0.4% EtOH (red), 2 weeks old aloin (purple). The measurements with EtOH were performed as a control. Fibrillation conditions A-C: ThT assay pH 7.4. Fibrillation conditions D: ThT assay pH 1.8.
(PDF)

S2 Fig Fibrillation kinetics of insulin incubated with aloin in the presence of anti-oxidants. Normalized fibrillation curves of 1 mg/mL (172 μM) insulin in the presence of (A) 0.4% EtOH (red), 400 μM aloin + 0.1% sodium azide stored in solution for 0 (gray), 1 (blue), 2 (green) and 3 (black) weeks, (B) 0.4% EtOH (red), 2.66 mM ascorbic acid (brown), 400 μM aloin + 2.66 mM ascorbic acid stored in solution for 0 (gray), 1 (blue), 2 (green) and 3 (black) weeks (C) 0.4% EtOH (red), 400 μM aloin stored in the dark for 0 (gray), 1 (blue) and 3 (black) weeks. Fibrillation conditions: ThT assay, pH 7.4.
(PDF)

S3 Fig Fractionation of aged aloin. (A) Fibrillation curves of 1 mg/mL (172 μM) insulin in the presence of 400 μM 2 weeks old aloin (green) or fractions hereof. Fibrillation conditions: ThT assay, pH 7.4. (B) Activity of processed aloin. Normalized fibrillation curves of insulin in the presence of 0.4% EtOH (red), 400 μM 2 weeks old aloin (green), dried and reconstituted 400 μM 2 weeks old aloin (blue), 400 μM fractionated, pooled, dried and reconstituted 2 weeks old aloin (black). Fibrillation conditions: pH 7.4.
(PDF)

S4 Fig Fibrillation kinetics of insulin incubated in the presence of degradation products of aloin. Normalized fibrillation curves of insulin in the presence of (A) 2% DMSO (green), 400 μM aloin in DMSO (black) and 400 μM aloin heated 10 minutes in DMSO (blue). (B) 0.4% EtOH (red), aloin incubated 1 week in PBS buffer (blue), (C) 2% DMSO (green), 400 μM aloe-emodine (blue) or 400 μM D-glucose (black), (D) 2% DMSO (green), 2% CH_2_Cl_2_ (purple), the CH_2_Cl_2_ phase of a 3 weeks old 400 μM aloin solution incubated with CH_2_Cl_2_ (blue) and the dried CH_2_Cl_2_ phase reconstituted in DMSO (black). Fibrillation conditions: ThT assay, pH 7.4.
(PDF)

S5 Fig C-13 NMR-spectra. C-13 NMR-spectra (300 K, 125 MHz) of aloin dissolved in methanol-*d*_4_ and D_2_O (1:4) during four weeks of storage at room temperature. (A) Overview 40–200 ppm, (B) 130–165 ppm, (C) 40–86 ppm, (D) expansion of signals from C-1 and C-8 (both hydroxylated aromatic carbons). (E) Expansion of signal from C-1’ (Glc C-1). Spectra were calibrated and normalized to the residual methanol signal at 49.15 ppm.
(PDF)

S6 Fig H-1 NMR-spectra. H-1 NMR-spectra (300 K, 600 MHz) of aloin dissolved in methanol-*d*_4_ and D_2_O (1:4) during four weeks of storage at room temperature. (A) 6.4–7.6 ppm (aromatic protons), (B) 2.7–4.7 ppm (non-aromatic protons), (C) 7.40±0.06 ppm (H-6), (D) 4.57±0.06 ppm (H-11a and H-11b, CH_2_-group). (E) 3.85±0.06 ppm (H-10). (F) 3.35±0.06 ppm (H-6’, low field signal of the diastereotopic protons in the CH_2_-group of glucose). Spectra were calibrated to the (suppressed) water signal set to 4.70 ppm and normalized to the range 6.2–7.7 ppm (all aromatic signals).
(PDF)
