# Supplementary material for: The Inhibitory Effect of Natural Products on Protein Fibrillation May Be Caused by Degradation Products – A Study Using Aloin and Insulin
Source: PLoS One. 2016 Feb 16;11(2):e0149148. doi: 10.1371/journal.pone.0149148 (PMC4755604; doi:10.1371/journal.pone.0149148)
Supplement: S1 Fig — Fibrillation curves of 1 mg/mL (172 μM) insulin in the presence of (A) 0.4% EtOH (red), 400 μM fresh aloin (blue), (B) 0.4% EtOH (red), 400 μM aloin stored in solution for 0 (blue), 1 (green), 2 (purple) and 3 (black) weeks, (C) 0.4% EtOH (red), 50 μM (gray), 100 μM (blue), 200 μM (purple), 400 μM (black) or 800 μM (green) 3 weeks old aloin, (D) 0.4% EtOH (red), 2 weeks old aloin (purple). The measurements with EtOH were performed as a control. Fibrillation conditions A-C: ThT assay pH 7.4. Fibrillation conditions D: ThT assay pH 1.8. (PDF) [file pone.0149148.s001.pdf]

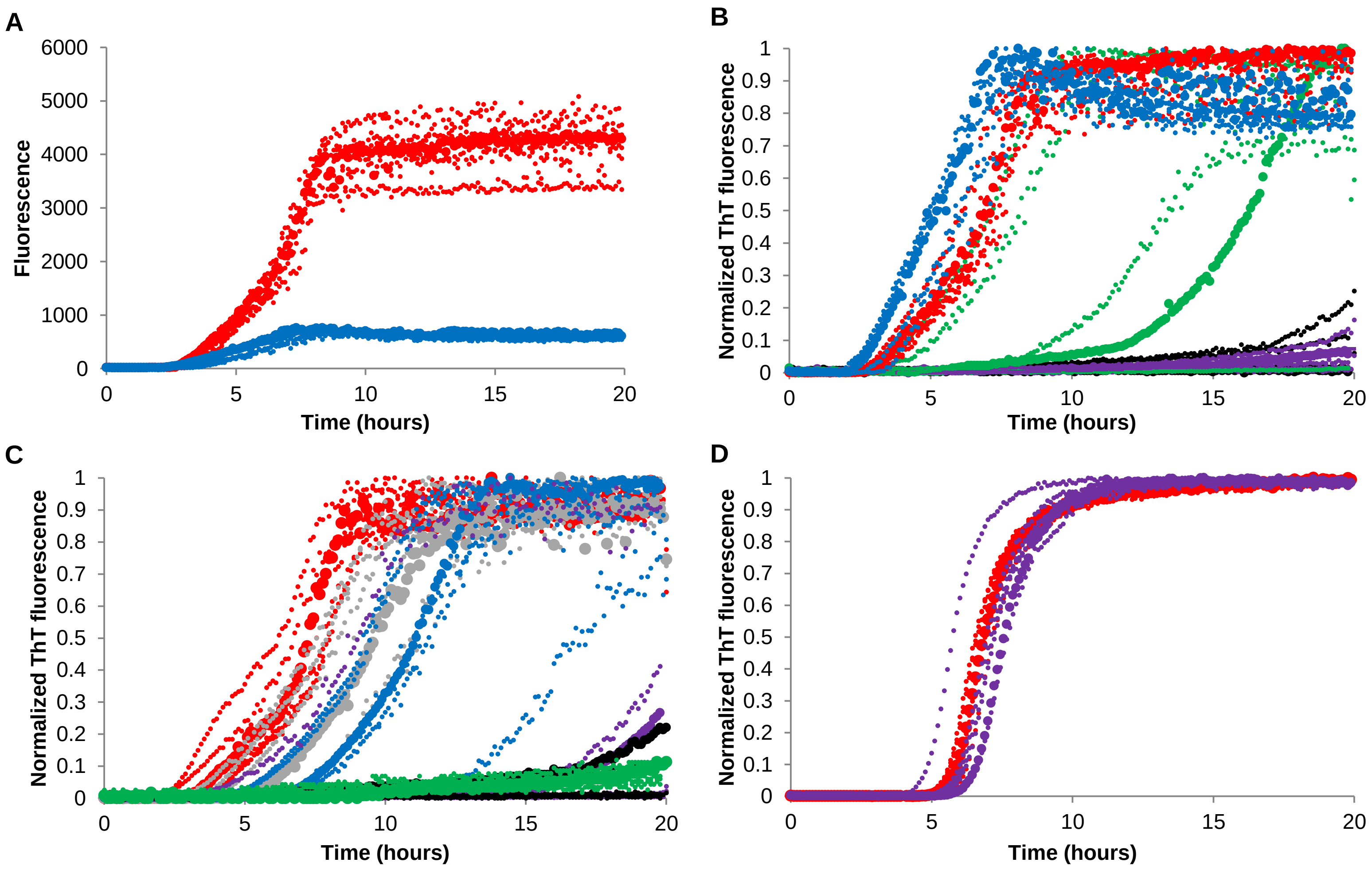

Fig S1. Fibrillation kinetics of insulin incubated in the presence of aged aloin. Fibrillation curves of 1 mg/mL (172 µM) insulin in the presence of (A) 0.4 % EtOH (red), 400 µM fresh aloin (blue), (B) 0.4 % EtOH (red), 400 µM aloin stored in solution for 0 (blue), 1 (green), 2 (purple) and 3 (black) weeks, (C) 0.4 % EtOH (red), 50 µM (gray), 100 µM (blue), 200 µM (purple), 400 µM (black) or 800 µM (green) 3 weeks old aloin, (D) 0.4 % EtOH (red), 2 weeks old aloin (purple). The measurements with EtOH were performed as a control. Fibrillation conditions A-C: ThT assay pH 7.4. Fibrillation conditions D: ThT assay pH 1.8.
