# Supplementary material for: The Inhibitory Effect of Natural Products on Protein Fibrillation May Be Caused by Degradation Products – A Study Using Aloin and Insulin
Source: PLoS One. 2016 Feb 16;11(2):e0149148. doi: 10.1371/journal.pone.0149148 (PMC4755604; doi:10.1371/journal.pone.0149148)
Supplement: S2 Fig — Normalized fibrillation curves of 1 mg/mL (172 μM) insulin in the presence of (A) 0.4% EtOH (red), 400 μM aloin + 0.1% sodium azide stored in solution for 0 (gray), 1 (blue), 2 (green) and 3 (black) weeks, (B) 0.4% EtOH (red), 2.66 mM ascorbic acid (brown), 400 μM aloin + 2.66 mM ascorbic acid stored in solution for 0 (gray), 1 (blue), 2 (green) and 3 (black) weeks (C) 0.4% EtOH (red), 400 μM aloin stored in the dark for 0 (gray), 1 (blue) and 3 (black) weeks. Fibrillation conditions: ThT assay, pH 7.4. (PDF) [file pone.0149148.s002.pdf]

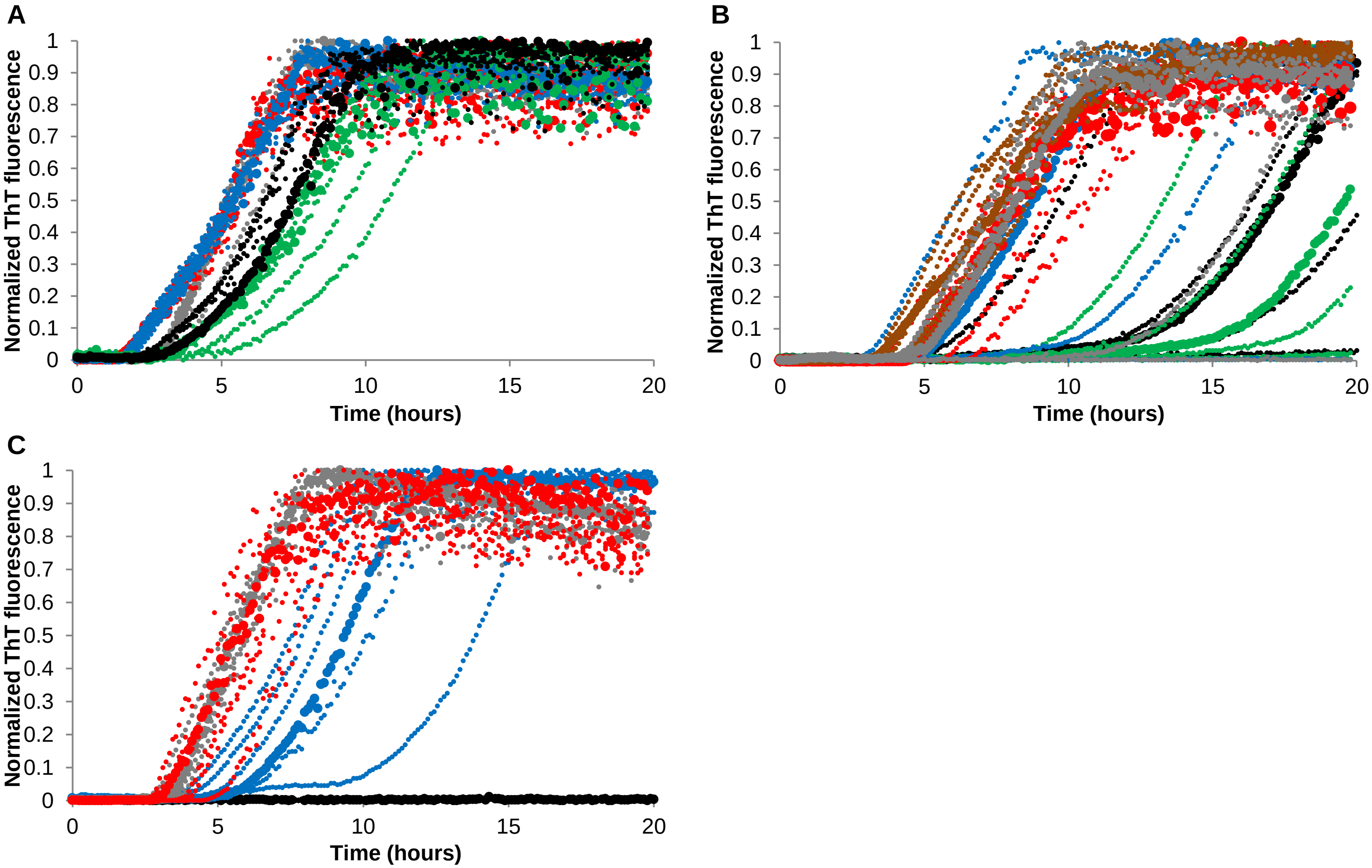

Fig S2. Fibrillation kinetics of insulin incubated with aloin in the presence of anti-oxidants. Normalized fibrillation curves of 1 mg/mL (172 µM) insulin in the presence of (A) 0.4 % EtOH (red), 400 µM aloin + 0.1 % sodium azide stored in solution for 0 (gray), 1 (blue), 2 (green) and 3 (black) weeks, (B) 0.4 % EtOH (red), 2.66 mM ascorbic acid (brown), 400 µM aloin + 2.66 mM ascorbic acid stored in solution for 0 (gray), 1 (blue), 2 (green) and 3 (black) weeks (C) 0.4 % EtOH (red), 400 µM aloin stored in the dark for 0 (gray), 1 (blue) and 3 (black) weeks. Fibrillation conditions: ThT assay, pH 7.4.
