# Supplementary material for: The Inhibitory Effect of Natural Products on Protein Fibrillation May Be Caused by Degradation Products – A Study Using Aloin and Insulin
Source: PLoS One. 2016 Feb 16;11(2):e0149148. doi: 10.1371/journal.pone.0149148 (PMC4755604; doi:10.1371/journal.pone.0149148)
Supplement: S3 Fig — (A) Fibrillation curves of 1 mg/mL (172 μM) insulin in the presence of 400 μM 2 weeks old aloin (green) or fractions hereof. Fibrillation conditions: ThT assay, pH 7.4. (B) Activity of processed aloin. Normalized fibrillation curves of insulin in the presence of 0.4% EtOH (red), 400 μM 2 weeks old aloin (green), dried and reconstituted 400 μM 2 weeks old aloin (blue), 400 μM fractionated, pooled, dried and reconstituted 2 weeks old aloin (black). Fibrillation conditions: pH 7.4. (PDF) [file pone.0149148.s003.pdf]

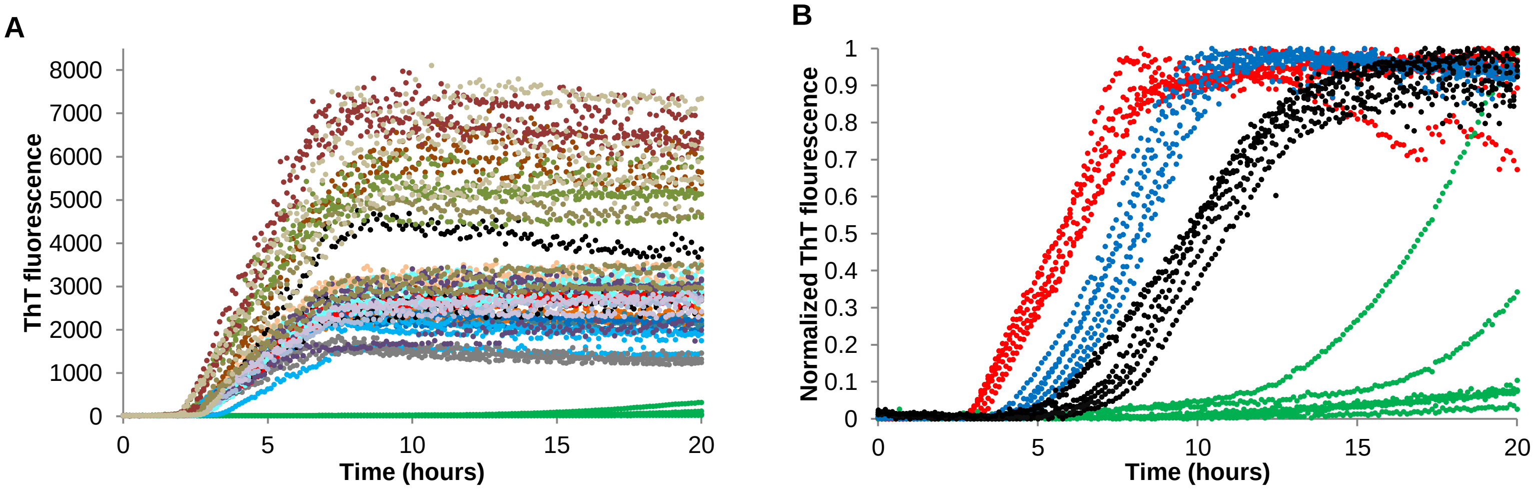


**Fig S3. Fractionation of aged aloin.** (A) Fibrillation curves of 1 mg/mL (172 µM) insulin in the presence of 400 µM 2 weeks old aloin (green) or fractions hereof. Fibrillation conditions: ThT assay, pH 7.4. (B) Activity of processed aloin. Normalized fibrillation curves of insulin in the presence of 0.4 % EtOH (red), 400 µM 2 weeks old aloin (green), dried and reconstituted 400 µM 2 weeks old aloin (blue), 400 µM fractionated, pooled, dried and reconstituted 2 weeks old aloin (black). Fibrillation conditions: pH 7.4.
