# Supplementary material for: The Inhibitory Effect of Natural Products on Protein Fibrillation May Be Caused by Degradation Products – A Study Using Aloin and Insulin
Source: PLoS One. 2016 Feb 16;11(2):e0149148. doi: 10.1371/journal.pone.0149148 (PMC4755604; doi:10.1371/journal.pone.0149148)
Supplement: S4 Fig — Normalized fibrillation curves of insulin in the presence of (A) 2% DMSO (green), 400 μM aloin in DMSO (black) and 400 μM aloin heated 10 minutes in DMSO (blue). (B) 0.4% EtOH (red), aloin incubated 1 week in PBS buffer (blue), (C) 2% DMSO (green), 400 μM aloe-emodine (blue) or 400 μM D-glucose (black), (D) 2% DMSO (green), 2% CH2Cl2 (purple), the CH2Cl2 phase of a 3 weeks old 400 μM aloin solution incubated with CH2Cl2 (blue) and the dried CH2Cl2 phase reconstituted in DMSO (black). Fibrillation conditions: ThT assay, pH 7.4. (PDF) [file pone.0149148.s004.pdf]

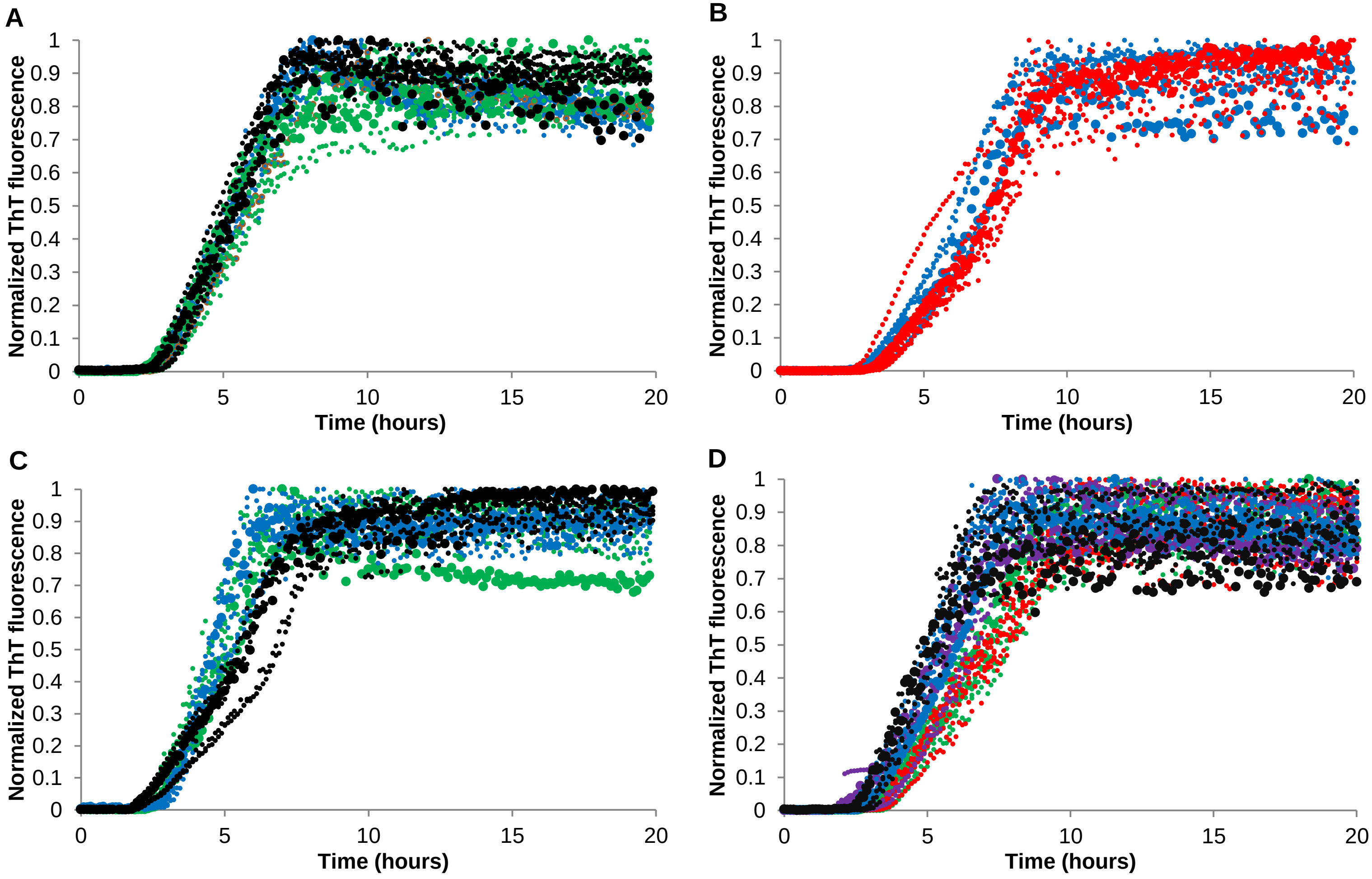

Fig S4. Fibrillation kinetics of insulin incubated in the presence of degradation products of aloin. Normalized fibrillation curves of insulin in the presence of (A) 2 % DMSO (green), 400 µM aloin in DMSO (black) and 400 µM aloin heated 10 minutes in DMSO (blue). (B) 0.4 % EtOH (red), aloin incubated 1 week in PBS buffer (blue), (C) 2 % DMSO (green), 400 µM aloe-emodine (blue) or 400 µM D-glucose (black), (D) 2 % DMSO (green), 2 % CH_2Cl_2 (purple), the CH_2Cl_2 phase of a 3 weeks old 400 µM aloin solution incubated with CH_2Cl_2 (blue) and the dried CH_2Cl_2 phase reconstituted in DMSO (black). Fibrillation conditions: ThT assay, pH 7.4.
