# Supplementary material for: The Inhibitory Effect of Natural Products on Protein Fibrillation May Be Caused by Degradation Products – A Study Using Aloin and Insulin
Source: PLoS One. 2016 Feb 16;11(2):e0149148. doi: 10.1371/journal.pone.0149148 (PMC4755604; doi:10.1371/journal.pone.0149148)
Supplement: S5 Fig — C-13 NMR-spectra (300 K, 125 MHz) of aloin dissolved in methanol-d4 and D2O (1:4) during four weeks of storage at room temperature. (A) Overview 40–200 ppm, (B) 130–165 ppm, (C) 40–86 ppm, (D) expansion of signals from C-1 and C-8 (both hydroxylated aromatic carbons). (E) Expansion of signal from C-1’ (Glc C-1). Spectra were calibrated and normalized to the residual methanol signal at 49.15 ppm. (PDF) [file pone.0149148.s005.pdf]

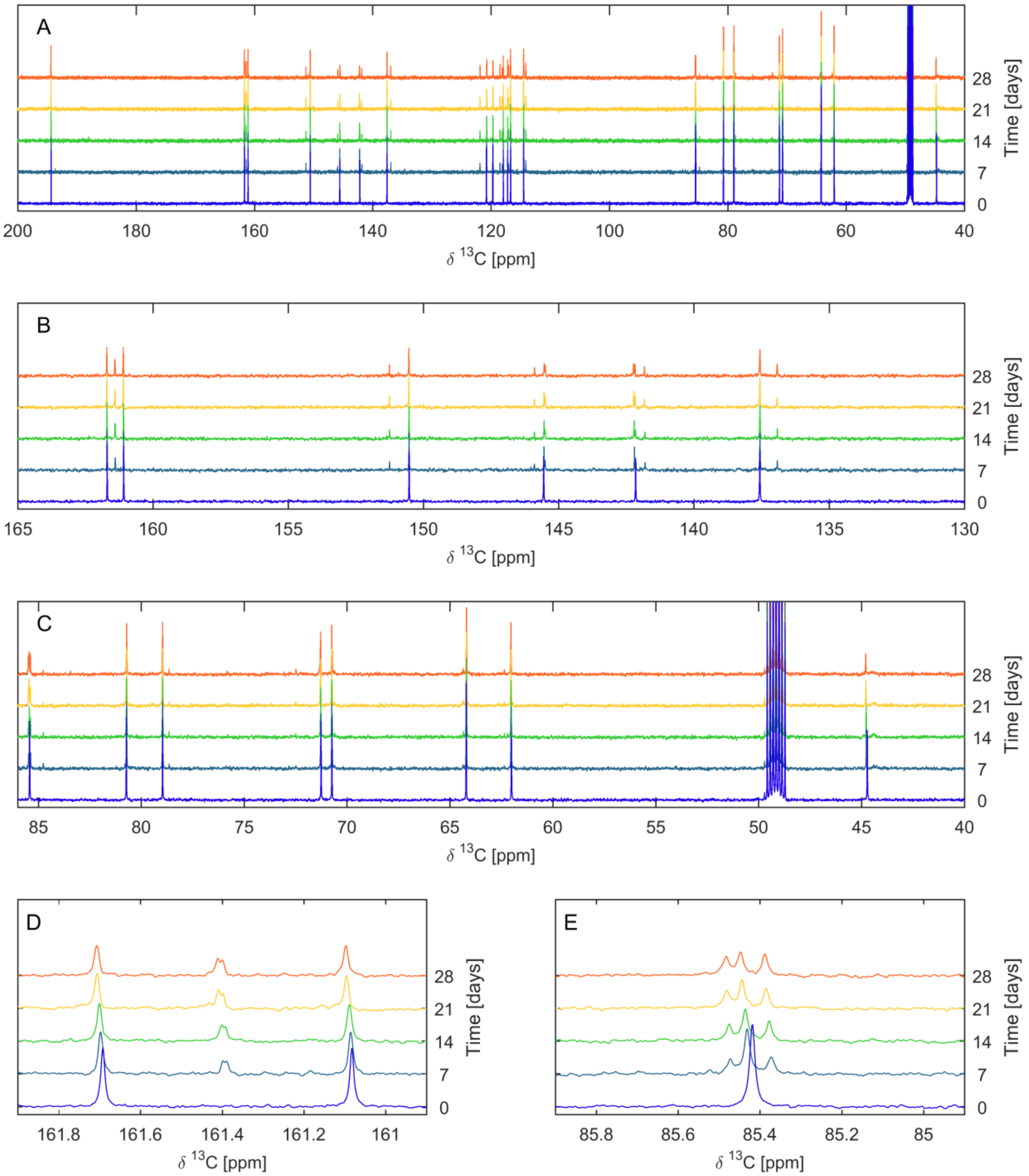

S5 Fig. C-13 NMR-spectra. C-13 NMR-spectra (300 K, 125 MHz) of aloin dissolved in methanol-d_4 and D_2O (1:4) during four weeks of storage at room temperature. (A) Overview 40-200 ppm, (B) 130-165 ppm, (C) 40-86

ppm, (D) expansion of signals from C-1 and C-8 (both hydroxylated aromatic carbons). (E) Expansion of signal from C-1’ (Glc C-1). Spectra were calibrated and normalized to the residual methanol signal at 49.15 ppm.
