# Supplementary material for: The Inhibitory Effect of Natural Products on Protein Fibrillation May Be Caused by Degradation Products – A Study Using Aloin and Insulin
Source: PLoS One. 2016 Feb 16;11(2):e0149148. doi: 10.1371/journal.pone.0149148 (PMC4755604; doi:10.1371/journal.pone.0149148)
Supplement: S6 Fig — H-1 NMR-spectra (300 K, 600 MHz) of aloin dissolved in methanol-d4 and D2O (1:4) during four weeks of storage at room temperature. (A) 6.4–7.6 ppm (aromatic protons), (B) 2.7–4.7 ppm (non-aromatic protons), (C) 7.40±0.06 ppm (H-6), (D) 4.57±0.06 ppm (H-11a and H-11b, CH2-group). (E) 3.85±0.06 ppm (H-10). (F) 3.35±0.06 ppm (H-6’, low field signal of the diastereotopic protons in the CH2-group of glucose). Spectra were calibrated to the (suppressed) water signal set to 4.70 ppm and normalized to the range 6.2–7.7 ppm (all aromatic signals). (PDF) [file pone.0149148.s006.pdf]

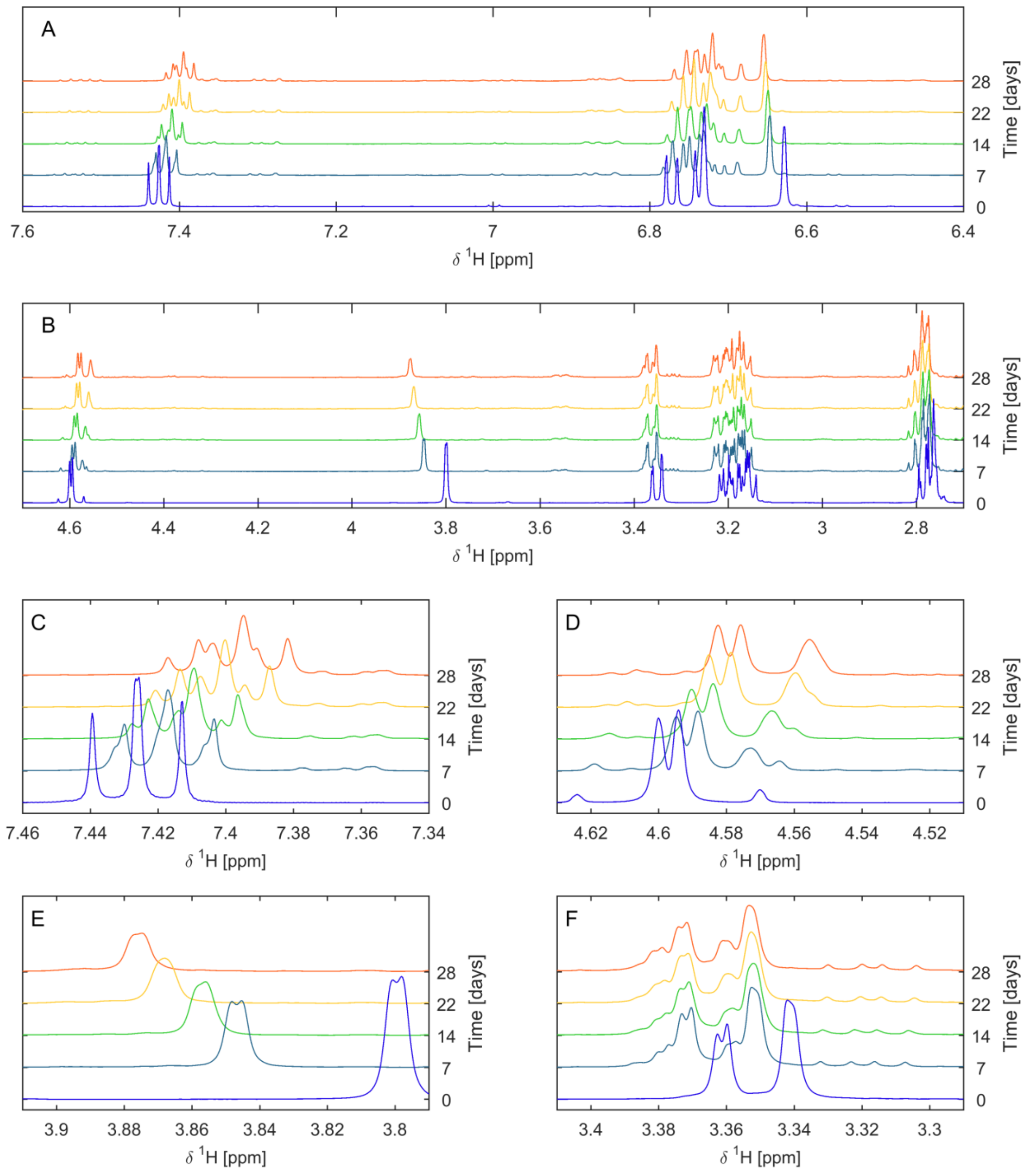


**S6 Fig. H-1 NMR-spectra.** H-1 NMR-spectra (300 K, 600 MHz) of aloin dissolved in methanol-$d_4$ and $D_2O$ (1:4) during four weeks of storage at room temperature. (A) 6.4-7.6 ppm (aromatic protons), (B) 2.7-4.7 ppm (non-aromatic protons), (C) 7.40±0.06 ppm (H-6), (D) 4.57±0.06 ppm (H-11a and H-11b, $CH_2$-group). (E)

3.85±0.06 ppm (H-10). (F) 3.35±0.06 ppm (H-6’, low field signal of the diastereotopic protons in the $CH_2$-group of glucose). Spectra were calibrated to the (suppressed) water signal set to 4.70 ppm and normalized to the range 6.2 – 7.7 ppm (all aromatic signals).
